# Micronutrients and ICU-Acquired Weakness: A Narrative Review

**DOI:** 10.3390/jcm15156066

**Published:** 2026-08-04

**Authors:** Alberto Nicolò Galvano, Artemide Maria Di Nicolò, Roberto Cusimano, Antonino Giarratano, Andrea Cortegiani

**Affiliations:** 1 Department of Precision Medicine in Medical, Surgical and Critical Care (Me.Pre.C.C.), University of Palermo, 90127 Palermo, Italy; 2 Department of Anesthesia, Analgesia, Intensive Care and Emergency, University Hospital Policlinico “Paolo Giaccone”, 90127 Palermo, Italy

**Keywords:** micronutrients, critical care, Intensive Care Unit-Acquired Weakness, critical illness myopathy, critical illness polyneuropathy, Post-Intensive Care Syndrome, muscle weakness, trace elements, vitamins

## Abstract

Intensive Care Unit-Acquired Weakness (ICU-AW) is a secondary neuromuscular disorder diagnosed during a critical illness and related to ICU stay and intensive treatments, once other possible etiologies have been excluded. Micronutrients are essential components of human nutrition that are needed in small amounts to maintain pivotal organ functions, but their role in critical care nutrition and in prevention and treatment of ICU-AW is unclear. This narrative review aimed to provide a synthesis of the current evidence on the potential role of micronutrients in ICU-AW, with a glance at what is known in medical settings outside the ICU. Available evidence is fragmented, showing controversial results. Studies on vitamin K1 and vitamin D suggest potential benefits on handgrip strength, skeletal muscle mass and mobility, while Coenzyme Q10 may have a role in improving skeletal muscle composition. Moreover, zinc homeostasis may influence muscle degradation, whereas copper levels may represent a risk factor. Evidence on iron and carnitine showed uncertain results. Conversely, more robust evidence on muscle weakness is available for medical settings outside the ICU. Larger observational studies and RCTs are needed to further evaluate the possible efficacy of micronutrient supplementation in the critical care setting.

## 1. Introduction

Intensive Care Unit-Acquired Weakness (ICU-AW) is defined as a secondary neuromuscular disorder developing during ICU stay, with no etiological explanation other than critical illness itself and its treatments [1,2]. It is estimated that around 25% of patients requiring prolonged mechanical ventilation may develop global and persistent weakness, meaning that up to 1 million patients worldwide are at risk of developing ICU-AW annually [1].

Micronutrients are essential components of human nutrition, needed in small daily or monthly amounts yet with a critical impact on health [3] through many physiological processes [4]. Vitamin deficiencies at the time of admission are common in ICU patients [5], highlighting a relationship between degree of inflammation and out-of-range vitamin levels. These findings may reflect increased consumption, as well as losses occurring during hospitalization [4,5,6]. Moreover, patients with multiple vitamin deficiencies seem to be at higher risk of longer hospitalization [5].

Inadequate provision of micronutrients during ICU stay, especially during the acute phase of critical illness, may further contribute to micronutrient deficiency [7]. As the micronutrient content of commercially available formulas depends on the volume administered, patients receiving less than the target volume may not achieve recommended micronutrient provision, and additional enteral or parenteral administration may be considered [7,8].

Gastrointestinal dysfunction is frequent in critically ill patients and may reduce effective nutrient delivery through feeding intolerance and interruption of enteral nutrition, while increased gastrointestinal losses may contribute to nutrient depletion [9]. Nevertheless, limited clinical evidence suggests that certain enterally administered micronutrients may increase their circulating concentration during critical illness, suggesting that intestinal uptake may be at least partly preserved [10,11]. However, these studies did not directly evaluate fractional absorption or specifically patients with gastrointestinal dysfunction. Therefore, the effect of critical illness on micronutrient absorption remains incompletely characterized.

Specific knowledge on micronutrients remains limited among clinicians, with trace elements being less well known than vitamins [8]. For the ICU setting, a recent survey by the European Society of Intensive Care Medicine (ESICM) has demonstrated a wide variation in current practices, potentially representing a risk for inadequate prevention, diagnosis, or treatment of micronutrient deficiency [4]. For example, only 35.6% of the respondents affirmed to adopt a protocol for parenteral micronutrient administration [4].

This issue had already been partially addressed by the European Society of Parenteral and Enteral Nutrition (ESPEN), releasing dedicated guidelines for clinicians [8] and nutritional recommendations for ICU patients [7].

Recently, increasing attention has been directed toward micronutrient status, assessment, and supplementation in critically ill patients, with growing emphasis on individualized approaches [8,12,13,14,15]. Although recent reviews have broadly addressed these topics, the specific relationship between micronutrients and muscle weakness, particularly ICU-acquired weakness, has not been their primary focus.

At the same time, a growing scientific interest has been registered in developing an appropriate strategy for preventing Post-Intensive Care Syndrome, which includes ICU-AW [16,17]. Accordingly, the aim of this narrative review was to summarize the available direct and indirect evidence on the role of micronutrients in the development, prevention, or treatment of muscle weakness related to intensive care unit admission and to provide new perspectives for future research.

## 2. Materials and Methods

For the purposes of this review, a structured, non-systematic literature search was conducted using the PubMed database. The search strategy included a combination of key terms and MeSH terms related to critical illness and intensive care, individual micronutrients, and ICU-acquired weakness-related terms such as sarcopenia and muscle weakness. The complete search string is provided as Supplementary Material S1. Additional studies were identified using the PubMed “Similar articles” function and by screening the reference lists of relevant articles. The micronutrients considered in this review were those addressed in the ESPEN micronutrient guideline, consistent with the WHO definition of micronutrients. Original articles on adult patients published in English were selected if pertinent to micronutrient status, deficiency, or supplementation in relation to ICU-acquired weakness or relevant muscle-related functional outcomes, including muscle mass, muscle strength, physical function, and sarcopenia. Randomized controlled trials, observational studies, and relevant preclinical studies were included and used by the authors to provide a narrative synthesis. A summary of the studies included is available in Table 1.

Each micronutrient-specific section was organized according to a consistent structure comprising its physiological functions, the causes of deficiency, the available evidence identified during the literature search, and the ESPEN guidance for critically ill patients. A summary of this guidance is available in Table 2.

To provide a broader biological and clinical context, evidence concerning the relationship between micronutrients and muscle weakness outside the ICU setting was discussed separately. This section was not derived from the structured PubMed search described above and was developed as a narrative overview based on relevant original studies and reviews.

## 3. Intensive Care Unit-Acquired Weakness

ICU-AW refers to the clinical diagnosis of a generalized, symmetrical neuromuscular dysfunction arising after ICU admission, which cannot be attributed to other causes [2]. It usually involves the limbs, the proximal more than the distal, and the respiratory muscles, while sparing facial and ocular muscles [2]. Furthermore, muscle tone and muscle strength appear to be reduced, with deep tendon reflexes being reduced or normal [2]. Loss of muscle mass is invariably present, although it may have a different etiology in the absence of electrophysiological abnormalities [29]. Another symptom occurring in patients with ICU-AW is muscle fatigue [30], which may potentially contribute to fatigue in ICU survivors [31].

ICU-AW is a common complication in the critical care setting, and it is related to longer ICU stay, prolonged mechanical ventilation, and higher ICU, in-hospital, and 1-year mortality [32]. The pathophysiology behind ICU-AW is still not clearly defined, but evidence suggests a complex interplay of pathophysiological mechanisms in response to critical illness and invasive treatments. Indeed, it may originate from a neurogenic disturbance, called “critical illness polyneuropathy”, a myogenic dysfunction, labeled “critical illness myopathy”, but it can also derive from a combination of them, defined “critical illness neuromyopathy” [2]. The clinical presentation may slightly vary according to the prevalent mechanism, but some features are common to all patients. Among these, ICU-AW is very frequently characterized by a profound muscle mass loss that can exceed 10% in the first week from admission in ICU [2].

Nutritional factors may play a role in these mechanisms, as muscle wasting in critical illness is characterized by enhanced catabolism and depressed anabolism, which may persist despite early caloric and protein provision [2,29]. Micronutrients have also been proposed as potential contributors, although their specific role in ICU-AW remains uncertain [33].

Muscle dysfunction and muscle mass loss contribute to functional impairment and, along with the other components, may affect patients long after hospital discharge [34].

**Figure 1:**
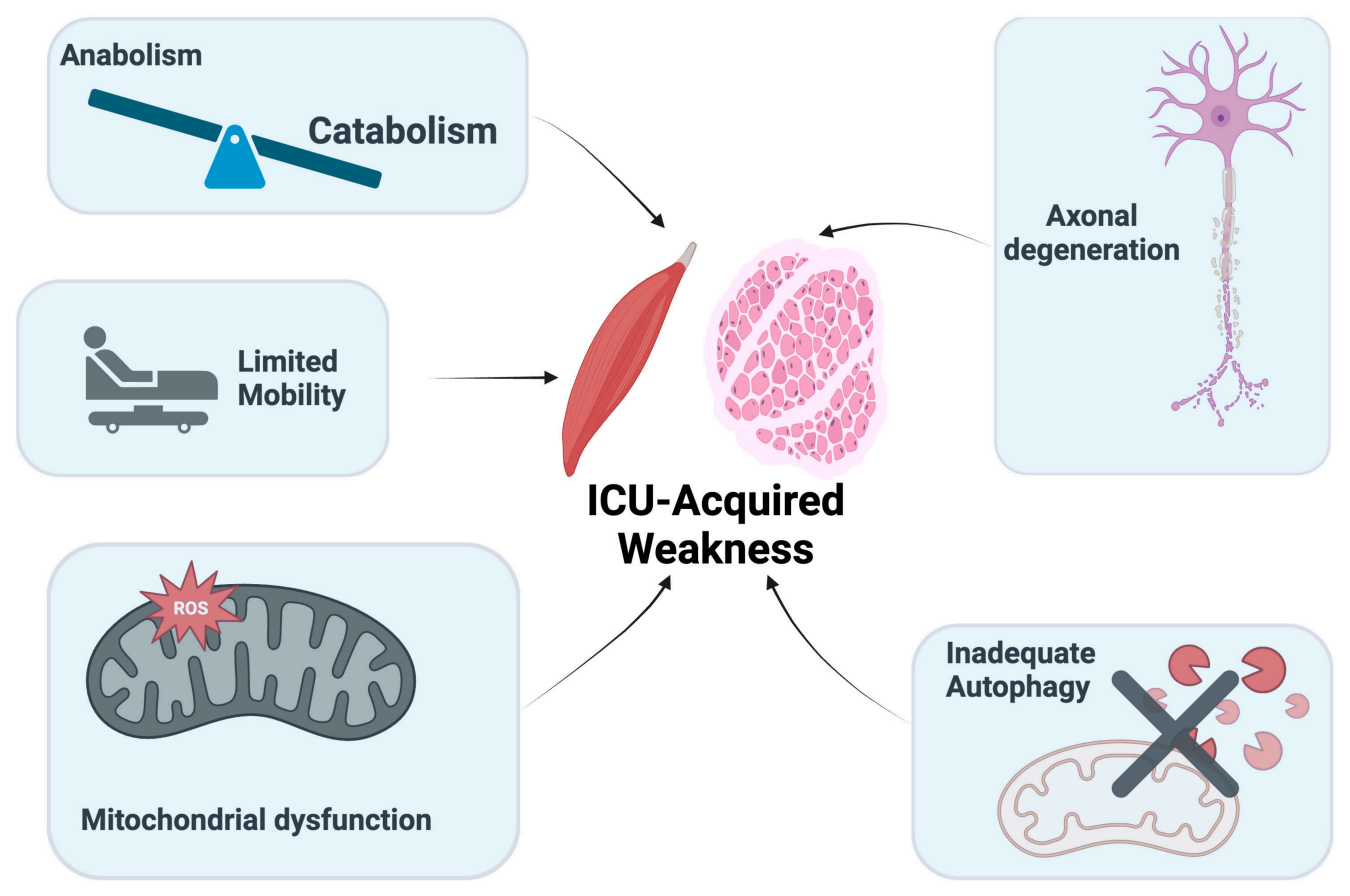
Framework of the major pathological mechanisms implicated in the development of ICU-acquired weakness. Various mechanisms contribute to the pathogenesis of muscle weakness, including impaired protein metabolism towards catabolism, limited mobility, mitochondrial dysfunction, axonal degeneration of motor neurons, and inadequate autophagy of damaged cytoplasmic organelles.

The main pathophysiological pathways are summarized in Figure 1.

### 3.1. Muscle Atrophy

Critical care patients dwell in a catabolic state strictly connected to their acute diseases, due to an imbalance between catabolic and anabolic effector hormones [2]. Furthermore, limited mobility due to ICU treatments results in pronounced muscle mass loss by unbalancing protein turnover towards proteolysis [2,30].

### 3.2. Bioenergetic Failure

Muscle weakness is also characterized by a loss of muscle function. Mitochondrial dysfunction seems to result from impaired oxygen utilization caused by direct mitochondrial damage [2]. Inflammation, hyperglycemia, and production of free radicals may worsen the scenario. Lastly, altered mitochondria amplify the damage by releasing free radicals and reactive oxygen species, establishing a vicious cycle of organelle damage [32].

### 3.3. Inadequate Autophagy

Another mechanism probably implicated in ICU-AW seems to be impaired autophagy activation. The autophagy-lysosomal system represents a quality-control mechanism for maintenance of muscle homeostasis, which may lead to muscle atrophy if unbalanced [32]. Insufficient activation of the autophagy system results in accumulation of damaged organelles and contributes to cellular degeneration [2].

### 3.4. Axonal Degeneration

Axonal degeneration has been linked to muscle weakness, although its pathophysiology remains unclear. Sepsis-induced microvascular changes may compromise endoneurium, allowing penetration of toxic metabolites and resulting in axonal death [30,32]. Direct toxic effects of hyperglycemia and mitochondrial dysfunction may contribute to the process of degeneration [32].

## 4. Micronutrients and ICU-Acquired Weakness

Micronutrients are defined by the World Health Organization (WHO) as vitamins and minerals needed by the body in small amounts, but with a critical impact on health [3]. The deficiency of any of them may cause severe and even life-threatening conditions, but it may also lead to less clinically evident symptoms [3]. Despite scientific attention growing towards nutrition in the ICU setting, micronutrients remain largely underestimated, with a wide variability in local clinical practice [4]. Furthermore, the lack of large observational or interventional studies contributes to the uncertainty on how to optimize micronutrient assessment in critically ill patients [4,35].

In the following sections, we discuss the available evidence on the association between micronutrients and ICU-AW and provide, for each micronutrient, a synthesis of the relevant ESPEN guidance for critically ill patients. A summary of the relevant ESPEN guidance is provided in Table 2.

### 4.1. Iron

#### 4.1.1. Physiological Functions and Deficiency

It is the most abundant micronutrient and it is needed for almost all pathways for energy and substrate metabolism (e.g., oxygen binding in hemoglobin, electron transport in cytochromes, cell proliferation and differentiation) [8]. Iron deficiency is the most common nutritional deficit, potentially impairing physical and cognitive functions [8].

Risk factors for deficiency include reduced intake or altered absorption, chronic disease, frequent blood sampling, and a history of major surgery, especially bariatric surgery [8].

#### 4.1.2. Main Findings

Lasocki et al. have been investigating iron deficiency (ID) and its correlation with clinical outcomes in the last decade [18,19,20]. In a prospective, multicenter observational study, 107 anemic patients discharged from ICU were followed up at 28 days and 6 months to assess the prevalence of iron deficiency, fatigue, and muscle weakness [18]. The results of this study showed that ID and non-ID patients had similar prevalence of fatigue as well as a similar muscle weakness at ICU discharge. However, fatigue was higher in the ID-patients group, independently of Hb levels, at day 28 of follow-up.

Another study of 1161 patients from the FROG-ICU study, a multicentric prospective observational study, has demonstrated that iron deficiency may also be diagnosed by assessing hepcidin levels, and that severe ID may be associated with poor physical recovery at one-year follow-up after discharge from ICU [19].

To further evaluate these findings, a single-blinded RCT on the topic has been conducted on 399 anemic adult critically ill patients [20]. Iron deficiency was diagnosed based on hepcidin levels, and patients in the intervention arm were distinguished in functional and absolute ID and treated accordingly. However, no significant difference between the two groups was found in post-ICU length of stay or fatigue at day 30 of follow-up.

In 2025, Li Zhang et al. conducted a Mendelian randomization (MR) to assess the potential impact of certain micronutrients on ICU-AW [21]. With regard to iron, MR results suggested a causal association with acquired weakness in critically ill patients, supporting a possible protective role of iron [21].

#### 4.1.3. ESPEN Clinical Guidance

To date, evidence suggests investigating iron status in case of anemia or in case of persistent major fatigue [8]. Iron status should be assessed using a combination of plasma iron, transferrin, transferrin saturation, ferritin, CRP, hepcidin, and red blood cell morphology. Anemic, critically ill patients with iron deficiency, as confirmed by hepcidin levels, should receive supplementation with a carbohydrate product of 1 g of iron [8]. Enteral nutrition should provide 18–30 mg/day of iron, whereas parenteral nutrition should implement at least 1 mg/day to avoid storage depletion [8].

### 4.2. Zinc

#### 4.2.1. Physiological Functions and Deficiency

Zinc is an essential trace element involved in many enzymatic reactions and metabolic pathways [8]. Zinc is mainly stored in skeletal muscle and bone, and only a small fraction circulates in plasma, mainly bound to albumin [8].

In addition to genetic disorders (e.g., Acrodermatitis enteropathica), risk factors for acquired deficiency include inadequate intake or impaired absorption, and increased gastrointestinal loss, for example due to enterostomy. Urinary losses may occur during hypercatabolic conditions, such as sepsis, burns, and trauma, while prolonged renal replacement therapy may cause extracorporeal losses [8].

Furthermore, Zinc levels commonly decrease as part of the acute-phase response in critical illness and usually recover with clinical improvement [8].

#### 4.2.2. Main Findings

The role of zinc in Critical Illness Myopathy (CIM) was investigated by Ribeiro et al. [22] by performing analysis on serial muscle biopsies and blood collections on a cohort of 7 neuroICU patients, together with a murine experimental model exposed to ICU conditions (mechanical ventilation and immobilization). The study showed that CIM was associated with significant disturbances in zinc homeostasis and muscle protein degradation, with a preferential loss of myosin (i.e., the hallmark of CIM) [22]. In parallel, genes involved in muscle regeneration were also downregulated [22].

In contrast, results on zinc from the MR analysis performed by Li Zhang et al. did not support a causal association between this element and ICU-AW [21].

#### 4.2.3. ESPEN Clinical Guidance

According to ESPEN, plasma zinc should be measured in critically ill patients with increased gastrointestinal or cutaneous losses and interpreted together with CRP and albumin [8]. Enteral nutrition should provide at least 10 mg/day per 1500 kcal, whereas parenteral nutrition should provide 3–5 mg IV/day in patients without abnormal losses [8]. In patients receiving parenteral nutrition who remain nil by mouth and have ongoing gastrointestinal losses, doses up to 12 mg IV/day may be required for as long as these losses persist. Patients with major burns involving >20% of the body surface area should receive 30–35 mg IV/day for 2–3 weeks [8].

### 4.3. Vitamin D

#### 4.3.1. Physiological Functions and Deficiency

Vitamin D may be defined as a steroid hormone precursor. The main production is endogenous, and it is mediated by exposure to UV-B rays, while dietary intake has a limited role and does not cover usual human needs [8]. Vitamin D is involved in bone and muscle metabolism, inflammation, immune and cardiovascular systems, and neuropsychiatric functions [36]. Risk factors for deficiency include severe kidney or liver dysfunction, chronically ill patients, and prolonged immobilization [8].

Definition and relevance of vitamin D deficiency remain a matter of debate in the acutely ill patient [8]. Mild to moderate deficiency is mainly asymptomatic, while severe forms may manifest as a variety of symptoms, including osteomalacia, muscle weakness, tetany, hypocalcemia, and a weakened immunological system [37].

#### 4.3.2. Main Findings

Muscle dysfunction in septic patients and vitamin D has been evaluated in a monocentric, prospective observational study [23]. In this study, muscle mass of 45 patients has been evaluated with ultrasound visualization of rectus femoris cross-sectional area and assessed until hospital discharge. Handgrip strength and MRC score were used to evaluate muscle strength. Interestingly, vitamin D levels were increased on day 2, at ICU discharge, and at hospital discharge compared with those registered at admission, with a positive association with handgrip strength.

The relation between vitamin D levels and in-hospital complications and morphofunctional recovery has been investigated in a prospective observational study by Simon-Frappoli et al. [24]. In a cohort of ICU patients admitted due to severe COVID pneumonia, vitamin D serum levels, functional tests, and body composition were assessed after hospital discharge and then reassessed after a six-month follow-up program. Vitamin D supplementation was prescribed in case of deficiency. The study reported a high prevalence of low vitamin D levels among patients recovering from severe COVID-19 [24]. Furthermore, those with deficient vitamin D levels experienced longer hospital and ICU stays, required longer invasive mechanical ventilation, and showed higher levels of systemic inflammation compared to those with sufficient vitamin D levels [24]. Low vitamin D levels (i.e., vitamin D < 30 ng/mL) were also associated with worse functional performance, while patients with sufficient levels showed better recovery during the six-month follow-up, with greater improvements in skeletal muscle index, right handgrip strength, and mobility [24].

#### 4.3.3. ESPEN Clinical Guidance

According to ESPEN guidelines, vitamin D status should be assessed in patients at risk of depletion or deficiency by measuring serum 25-hydroxyvitamin D [8]. Enteral nutrition should provide at least 1000 IU (25 μg) per day with 1500 kcal, whereas parenteral nutrition should provide at least 200 IU (5 μg) per day [8]. The frequency of monitoring should be individualized, and an additional amount of cholecalciferol should be administered in case of recurrent deficiency [8].

### 4.4. Vitamin K1

#### 4.4.1. Physiological Functions and Deficiency

Vitamin K is a group of lipid-soluble molecules needed as cofactors for so-called vitamin K-dependent proteins, involved in many functions for bone and vascular health, potentially also having an antioxidant effect [8]. Its vitamers are produced by plants (vitamin K1) or synthesized by human intestinal microbiota (vitamin K2) [8]. Most common causes of deficiency are connected to fat malabsorption (e.g., celiac disease, short bowel), but also malnutrition, antibiotics, and anticoagulation treatments may be involved [8].

#### 4.4.2. Main Findings

The role of vitamin K1 in sepsis-induced muscle weakness has been recently evaluated in a murine model [25]. In this study, mice were injected intraperitoneally with lipopolysaccharide and then treated by gavage with vitamin K1. Interestingly, results have shown that vitamin K1 attenuated sepsis severity, possibly by regulating the inflammatory response, and increased the grip strength of the treated group. Similarly, supplementation of vitamin K1 showed a protective effect on muscle damage, possibly by regulating oxidative stress and inflammatory response.

#### 4.4.3. ESPEN Clinical Guidance

As there is no agreed standard, vitamin K assessment should be obtained in at-risk patients, using a combination of biomarkers and dietary intake [8]. According to ESPEN micronutrients guidelines, enteral nutrition should provide at least 120 µg/day of vitamin K1, whereas parenteral nutrition should provide 150 µg/day [8].

### 4.5. Coenzyme Q10

#### 4.5.1. Physiological Functions and Deficiency

Coenzyme Q10 (CoQ10) is a fat-soluble molecule produced in the inner membrane of mitochondria [8]. Although it is not strictly considered a vitamin, it is included in the ESPEN guidelines on micronutrients for growing metabolic research [8]. It plays a pivotal role in mitochondrial bioenergetics, and it is also an endogenously produced antioxidant [8]. Its deficiency is usually determined by nutritional deficit of pyridoxine, an essential cofactor for its synthesis, but it may also derive from a defect in its production pathway [8]. Furthermore, plasma levels of CoQ10 may not be reliable to reflect actual availability in mitochondria due to increased loss via body fluids, increased endothelial permeability, inadequate intake, or increased needs [8].

#### 4.5.2. Main Findings

In a recent single-center RCT [26], 40 traumatic injury patients with subtherapeutic plasma levels of CoQ10 received a safe dose of CoQ10 supplementation to assess IL-6 concentration as the primary outcome. Among secondary outcomes, there were duration of mechanical ventilation, ICU and hospital length of stay, and body composition. After a short follow-up period, results showed a significant reduction in MV duration and ICU and hospital length of stay, with improvement in skeletal muscle composition with no significant changes in weight, arm circumference, and fat measurements.

#### 4.5.3. ESPEN Clinical Guidance

As there is no strong evidence available, ESPEN guidelines found no clinical reason to measure plasma levels of CoQ10, which may be evaluated only for research purposes [8].

### 4.6. Carnitine

#### 4.6.1. Physiological Functions and Deficiency

Carnitine has been included as an “assimilated vitamin” in the ESPEN micronutrients guidelines [8]. It is an amino acid derivative mainly found in high-energy-demanding tissues, such as skeletal muscles, myocardium, and liver, where it has the role of a carrier for transportation of long-chain fatty acids in beta-oxidation [8]. The acquired form of deficiency is more frequent in settings such as critical care. Indeed, critically ill patients are at high risk of deficiency, especially when malnourished, in treatment with continuous renal replacement therapy, or in prolonged parenteral nutrition without proper supplementation [8].

#### 4.6.2. Main Findings

In the 1990s, a single-center prospective observational study [27] assessed the prevalence of carnitine depletion in 72 critically ill patients with undernutrition. Unexpectedly, the results of the study showed a low prevalence (<5%) of carnitine deficit. Even though a reduced skeletal muscle mass may mirror a decrease in the carnitine pool, authors highlight the need for further investigation to determine whether supplementation may be of some benefit [27].

More recently, a secondary analysis of the EPaNIC RCT showed that both low and high carnitine concentrations were associated with prolonged ICU stay, longer need for life-support therapies, and increased 90-day mortality [28]. Furthermore, concentrations above 50 µmol/L were associated with a higher likelihood of developing ICU-AW and increased 2- and 5-year mortality [28].

#### 4.6.3. ESPEN Clinical Guidance

Current evidence is still insufficient to recommend routine assessment and addition of this element in enteral or parenteral nutrition [8]. In critically ill patients, it should be considered in the presence of unexpected loss of lean body mass accompanied by hypertriglyceridemia and hyperlactatemia, particularly during prolonged parenteral nutrition or continuous renal replacement therapy [8].

In patients with confirmed deficiency, L-carnitine may be administered at 2–5 mg/kg/day, using the same route as that used for macronutrient administration until biochemical normalization [8].

## 5. Micronutrients and Muscle Weakness: Indirect Evidence from Non-ICU Settings

To date, evidence available on the topic and in the setting of critical care appears fragmented and inconclusive due to the lack of large observational studies and RCTs.

However, a more extensive body of evidence is available for non-ICU settings. While not directly generalizable to ICU-acquired weakness, these findings provide important mechanistic insights and a useful framework for interpreting the available evidence in critically ill patients.

### 5.1. Selenium

#### 5.1.1. Physiological Functions and Deficiency

Selenium is an essential micronutrient, required for the synthesis of the amino acid selenocysteine, which constitutes selenoproteins [38]. They perform many biochemical functions, including regulation of thyroid hormone metabolism and control of cell proliferation and apoptosis [39]. Selenium is also required for the synthesis of the enzyme family of glutathione peroxidases, the first-line enzymes involved in antioxidant activity, therefore being involved as an anti-inflammatory agent [39]. Insufficient selenium intake is the most frequent cause of deficiency, and it can also occur in patients undergoing CRRT [40], burn patients [41], and those on long-term parenteral nutrition [8,42].

#### 5.1.2. Main Findings

Several studies have evaluated the possible role of selenium in muscle weakness. For example, a cross-sectional analysis of 891 individuals from the InCHIANTI study found that low plasma selenium levels were associated with poorer hip strength, knee strength, and grip strength [43].

Supplementation of selenium in the community-dwelling population has also been studied. In a randomized controlled trial, 443 individuals were invited to assume a combination of 200 mcg/day of organic selenium yeast and 200 mg/day of CoQ10, or placebo for 4 years. After 48 months, 206 participants were evaluated, highlighting that the experimental group had a lower decline in terms of mean change in relative scores in physical performance, vitality, and physical score compared to placebo [44].

#### 5.1.3. ESPEN Clinical Guidance

Considering the pathophysiology of selenium deficiency, ESPEN recommends measuring selenium status, together with CRP and albumin, in all patients undergoing parenteral nutrition for more than 2 weeks [8].

Enteral and parenteral nutrition should provide 50–150 μg/day per 1500 kcal and 60–100 μg/day, respectively [8]. A selenium plasma level below 0.4 µmol/L, or below 0.75 µmol/L in the absence of an inflammatory response, requires correction, starting with 100 μg/day using either the enteral or IV route [8].

### 5.2. Copper

#### 5.2.1. Physiological Functions and Deficiency

Copper is an essential trace element that acts mainly as a cofactor for many enzymes involved in fundamental biological processes [8]. It is crucial for the proper function of the nervous system, cardiovascular system, connective tissues, and cellular oxidative balance [8]. Some acute conditions may represent risk factors for copper depletion, such as major burns, gastric and bariatric surgery, renal replacement therapy, and prolonged artificial nutrition without adequate copper supplementation [8]. During inflammatory states, plasma copper concentrations tend to increase as the liver increases ceruloplasmin production. Thus, normal copper concentration in the presence of elevated C-Reactive Protein (CRP) may actually mask an underlying copper depletion or deficiency [8].

#### 5.2.2. Main Findings

In a cross-sectional observational study by Zhi Chen et al., the data of a cohort of 3860 people from the NHANES (National Health and Nutrition Examination Survey) database were analyzed using weighted multivariable linear regression models to investigate a possible association between serum copper levels and muscle mass, measured as appendicular skeletal muscle mass index (ASMI) [45]. Serum copper levels were also categorized into tertiles to compare ASMI across different exposure levels.

Interestingly, results found a negative association between serum copper levels and ASMI, with a threshold effect at approximately 150.6 μg/dL of serum copper [45].

#### 5.2.3. ESPEN Clinical Guidance

Plasma copper should be measured together with CRP in critically ill patients with unexplained neuropathy, major burns, continuous renal replacement therapy lasting more than 2 weeks, or previous abdominal surgery excluding the duodenum [8]. Enteral and parenteral nutrition should provide 1–3 mg/day per 1500 kcal and 0.3–0.5 mg/day, respectively [8]. Copper deficiency is likely when plasma concentrations are <12 μmol/L despite CRP > 20 mg/L, while values <8 μmol/L require repletion regardless of the inflammatory state. Severe deficiency should be treated intravenously with 4–8 mg/day administered as a slow infusion [8].

### 5.3. Vitamin B6

#### 5.3.1. Physiological Functions and Deficiency

It refers to a group of water-soluble pyridine compounds, termed B6 vitamers. Its biologically active form, pyridoxal phosphate, serves as a coenzyme for many enzymatic reactions [8]. Active vitamin B6 is involved in the biosynthesis and degradation of amino acids, in gluconeogenesis and neurotransmitter synthesis [8]. Populations at high risk of deficiency include alcoholics, patients on renal dialysis, critically ill patients, but also those receiving medical therapies that inhibit vitamin activity (i.e., corticosteroids, isoniazid) [8].

#### 5.3.2. Main Findings

Some studies have investigated a possible correlation of this vitamin with sarcopenia. For example, a cross-sectional analysis of 227 individuals from the Maastricht Sarcopenia Study has demonstrated that sarcopenic adults consumed less vitamin B6 than non-sarcopenic ones [46]. Furthermore, in a randomized controlled trial conducted on a murine model, consuming the RDA of vitamin B6 upregulated the expression of several protective genes involved in growth and repair of skeletal muscle [47].

#### 5.3.3. ESPEN Clinical Guidance

Vitamin B6 status should be assessed when deficiency is clinically suspected by measuring pyridoxal phosphate (PLP). In critically ill patients or in the presence of inflammation, red blood cell PLP is preferred [8].

ESPEN recommends delivering at least 1.5 mg/day of pyridoxine every 1500 kcal in enteral nutrition, while 4–6 mg/day is recommended in parenteral nutrition [8]. Additional amounts are indicated only in ascertained cases of isoniazid overdosing or glycol poisoning [8].

### 5.4. Vitamin B12

#### 5.4.1. Physiological Functions and Deficiency

Cobalamin is an essential water-soluble vitamin involved in numerous pathways as a cofactor of enzymes, including mitochondrial metabolism, immune response, maintenance of myelin sheath integrity, nucleic acid and neurotransmitter synthesis [8,31]. Its deficiency usually derives from inadequate intake, but intestinal malabsorption due to various causes (e.g., autoimmune or post-bariatric surgery) is also frequent [31]. Cobalamin deficiency requires several months to establish, as liver storage of approximately 2500 mcg requires up to 36 months of inadequate intake to empty. Physiological needs may increase in certain situations, such as age, increased oxidative stress, and use of certain medications [8].

#### 5.4.2. Main Findings

The relation between vitamin B12 and sarcopenia has been investigated in various observational studies. In a cross-sectional study matching 66 non-sarcopenic individuals to previously recruited 66 sarcopenic adults, it was found that the sarcopenic group had a lower dietary intake of vitamin B12 and lower vitamin B12 levels compared to controls [48].

Another study found that patients with levels of cobalamin lower than 400 pg/mL, compared to control patients, had lower lean body mass, total skeletal mass, and skeletal muscle mass index [49].

It is therefore possible that vitamin B12 may have a protective effect against the development of sarcopenia [50].

#### 5.4.3. ESPEN Clinical Guidance

Cobalamin deficiency should be investigated in patients with anemia, isolated macrocytosis, or established polyneuropathy. Assessment should combine at least two biomarkers, preferably holotranscobalamin and methylmalonic acid, with serum cobalamin used as an alternative marker [8]. Enteral and parenteral nutrition should provide at least 2.5 μg/day per 1500 kcal and 5 μg/day, respectively [8]. In acute symptomatic deficiency, pernicious anemia, total gastrectomy, or persistent malabsorption, 1000 μg should be administered intramuscularly every second day for 2 weeks (or daily for 5 days), while monitoring serum potassium [8].

### 5.5. Vitamin D

Various studies have been performed to assess the potential benefit of Vitamin D supplementation. In a recent randomized controlled trial, 115 individuals with vitamin D deficiency and sarcopenia received a nutritional supplement of 10.000 IU of cholecalciferol three times a week versus placebo [51]. Results showed improvement in appendicular skeletal muscle mass but not in grip strength after 6 months of follow-up compared to placebo [51]. Another RCT involving 160 postmenopausal women receiving 1000 IU of cholecalciferol daily versus placebo showed a significant increase in muscle strength of lower limbs compared to the control group after 9 months of follow-up [52].

### 5.6. Miscellanea: L-Carnitine and Coenzyme Q10

A recent review article on the role of nutritional factors in physical frailty (i.e., reduced physical activity, weak muscles, difficulty walking, decreased endurance, and unexplained weight loss) analyzed the role of CoQ10 and L-carnitine in modifying its course [53]. Regarding CoQ10, aging has been associated with a decline in its serum levels, and there seems to be an inverse correlation between serum CoQ10 levels and the degree of sarcopenia. In particular, CoQ10 deficiency has been linked to higher odds of sarcopenia and physical dysfunction. Furthermore, CoQ10 supplementation may contribute to improving or restoring motor function [53].

L-carnitine has been proposed as a potentially useful nutrient due to its role in energy production, fat metabolism, and reducing inflammation, which is strongly linked to muscle loss in aging [53].

However, current evidence remains inconsistent. While some studies reported improvements in frailty scores, handgrip tests, and overall physical performance after supplementation of L-carnitine, a double-blind, placebo-controlled RCT showed no significant improvements in muscle strength in healthy older women after a 24-week supplementation [53].

Thus, both nutrients appear promising in reducing sarcopenia, but more robust studies are needed [53].

## 6. Strengths and Limitations

A key strength of this narrative review is its focused examination of the potential role of micronutrients in ICU-acquired weakness. However, several limitations should be acknowledged. Consistent with its narrative design, the literature search was conducted in a structured but non-systematic manner and was limited to the PubMed database. Therefore, some relevant studies may not have been identified. Moreover, several studies evaluated related but non-equivalent muscle or functional outcomes. The available evidence was heterogeneous in terms of patient populations, outcome measures and study designs, and also included preclinical studies. Finally, when direct evidence from critically ill patients was limited or unavailable, evidence from non-ICU settings was also considered. Although these findings may provide useful biological and clinical context, they cannot be directly linked to the critical care setting.

## 7. Conclusions

Nutrition in critical care is pivotal, and its clinical practice is constantly evolving to achieve better outcomes. Micronutrients have been highlighted as essential elements to avoid the development or worsening of critical conditions in ICU patients, but little is known about when to measure, how much to administer, and when to supplement additional amounts. Although some evidence may suggest a beneficial role of therapeutic micronutrient supplementation, results are controversial. Large observational studies and RCTs in critical care settings are needed to further investigate this topic.

## Figures and Tables

**Table 1:** Main characteristics and findings of studies investigating micronutrients in relation to ICU-acquired weakness and related muscle outcomes.

Micronutrient	Study	Design and Population	Micronutrient Status or Intervention	Muscle-Related Outcome(s)	Main Findings
Iron	Lasocki et al., 2014 [18]	Prospective multicenter observational study; 107 anemic adults with ICU stay ≥5 days; follow-up at day 28 and 6 months	Iron status assessed at ICU discharge, day 28, and 6 months using ferritin and transferrin saturation	Handgrip strength at ICU discharge; fatigue assessed using a numerical scale and the MFI-20 at day 28 and 6 months	No differences in handgrip strength or fatigue at ICU discharge; iron deficiency was associated with greater fatigue at day 28, but not at 6 months
Iron	Lasocki et al., 2018 [19]	Prospective multicenter observational analysis of the FROG-ICU cohort; 1161 ICU survivors; 1-year follow-up	Iron status assessed at ICU discharge using hepcidin, ferritin, and the soluble transferrin receptor/log ferritin ratio	Physical quality of life at 1 year, assessed using the physical component of the SF-36	Severe iron deficiency was independently associated with poorer physical quality of life at 1 year
Iron	Lasocki et al., 2021 [20]	Single-blind RCT; 399 anemic critically ill adults with ICU stay ≥5 days	Intervention: hepcidin-guided treatment with ferric carboxymaltose 1 g IV for hepcidin <20 μg/L, or ferric carboxymaltose 1 g IV plus erythropoietin for hepcidin 20–<41 μg/L; control: standard care with blinded hepcidin measurement	Fatigue at day 30; post-ICU length of stay	Hepcidin-guided treatment did not improve post-ICU length of stay or fatigue at day 30
Iron	Zhang et al., 2025 [21]	Mendelian randomization study	Genetically predicted iron status	ICU-acquired weakness	Genetically predicted iron status showed a potential protective association with ICU-AW
Zinc	Ribeiro et al., 2024 [22]	Translational longitudinal study; 7 neuro-ICU patients exposed to 12 days of mechanical ventilation and immobilization with six serial muscle biopsies, plus rat models of CIM	Zinc transporter expression, intramuscular zinc levels, and activation of zinc-dependent MMPs assessed in skeletal muscle	Preferential myosin loss; MMP activation; muscle protein degradation	Altered zinc homeostasis and MMP activation accompanied preferential myosin loss; findings were reproduced in experimental models
Zinc	Zhang et al., 2025 [21]	Mendelian randomization study	Genetically predicted zinc status	ICU-acquired weakness	No causal association between genetically predicted zinc status and ICU-AW was identified
Vitamin D	Borges et al., 2020 [23]	Prospective single-center observational study; 45 patients with severe sepsis or septic shock	Serial serum vitamin D assessment during hospitalization	Rectus femoris muscle mass assessed by ultrasound; handgrip strength and MRC score after awakening	Vitamin D levels were positively associated with handgrip strength after awakening
Vitamin D	Simon-Frappoli et al., 2024 [24]	Prospective observational study; 94 postcritical COVID-19 patients assessed 2 weeks after hospital discharge and after a 6-month multidisciplinary follow-up program	Vitamin D status classified as sufficient, insufficient, or deficient; changes in vitamin D status assessed during follow-up	Skeletal muscle index; skeletal muscle mass-to-weight ratio; right-hand handgrip strength; timed up-and-go; 6 min walk test; body composition	Vitamin D deficiency was associated with poorer muscle status; normal levels were associated with greater morphofunctional recovery at 6 months
Vitamin K1	Xiao et al., 2024 [25]	Preclinical study; fecal microbiota transplantation and LPS-induced inflammation mouse models, followed by vitamin K1 intervention	Fecal and serum vitamin K1 levels; oral vitamin K1 administration by gavage after LPS exposure	Grip strength; skeletal muscle damage and atrophy; balance between muscle protein synthesis and catabolism	Vitamin K1 improved grip strength and attenuated muscle atrophy and damage in an LPS-induced sepsis model
Coenzyme Q10	Hasanloei et al., 2021 [26]	Single-center, randomized, placebo-controlled trial; 40 mechanically ventilated trauma ICU patients	Intervention: sublingual CoQ10, 400 mg/day for 7 days. Control: placebo	Fat-free mass; skeletal muscle mass; body cell mass; anthropometric measures; duration of mechanical ventilation; ICU and hospital length of stay	CoQ10 increased fat-free, skeletal muscle, and body cell mass. Reduced ventilation and length-of-stay findings should be interpreted cautiously
L-carnitine	Wennberg et al., 1992 [27]	Prospective single-center observational study; 60 critically ill patients with abnormal nutritional status or possible secondary carnitine deficiency, plus 12 healthy controls	Free and total carnitine measured in plasma and skeletal muscle; urinary carnitine excretion; no supplementation	Skeletal muscle carnitine content; body composition; no direct muscle-strength or ICU-AW assessment	Carnitine depletion occurred in <5% of patients; plasma and muscle carnitine concentrations were not correlated
L-carnitine	Lauwers et al., 2026 [28]	Secondary observational analysis of the EPaNIC RCT; 1600 patients with plasma free carnitine measured on ICU day 6	Plasma free carnitine categorized as low (<24 μmol/L), intermediate (24–50 μmol/L), or high (>50 μmol/L)	Muscle weakness; time to ICU discharge; duration of ICU and hospital dependency and life-support therapies; short- and long-term mortality	Low and high carnitine levels were associated with delayed ICU recovery and higher mortality; levels >50 μmol/L were also associated with muscle weakness

Abbreviations: CIM, critical illness myopathy; CoQ10, coenzyme Q10; ICU-AW, Intensive Care Unit-Acquired Weakness; LPS, lipopolysaccharide; MFI-20, Multidimensional Fatigue Inventory-20; MMP, matrix metalloproteinase; MRC, Medical Research Council; RCT, randomized controlled trial; SF-36, 36-Item Short Form Health Survey.

**Table 2:** ESPEN guidance on the assessment, monitoring, and supplementation of micronutrients in critically ill patients [8].

Micronutrient	Patients at Risk/Indications for Assessment	What to Monitor and When	ESPEN Guidance on Dosing and Supplementation
Iron	Anemia; persistent major fatigue; chronic disease; frequent blood sampling/dialysis; major or bariatric surgery; ongoing blood loss; reduced intake or absorption.	Plasma iron; transferrin; transferrin saturation; ferritin; CRP; hepcidin; red blood cell morphology. Reassess 8–10 weeks after treatment; earlier ferritin measurement after IV iron may be misleading.	EN: 18–30 mg/day with 1500 kcal. PN: ≥1 mg/day elemental iron or equivalent periodic dose. Deficiency: 1 g IV iron as a single dose over ~15 min; same regimen in anemic critically ill patients with low hepcidin. Adapt PN dose if body weight <40 kg; caution with additional supplementation during infection or hematologic malignancy.
Zinc	Increased gastrointestinal or cutaneous losses; fistulae, stomas, or diarrhea; malabsorption, short bowel, or bariatric surgery; burns, trauma, or sepsis; prolonged renal replacement therapy; long-term PN.	Plasma zinc with CRP and albumin. Measure when commencing long-term PN in patients with increased gastrointestinal or skin losses; repeat according to ongoing risk. Every 6–12 months during long-term PN.	EN: ≥10 mg/day with 1500 kcal. PN: 3–5 mg IV/day without abnormal losses. Ongoing GI losses while nil by mouth: up to 12 mg IV/day while losses persist. Major burns >20% BSA: 30–35 mg IV/day for 2–3 weeks. Acquired deficiency: 0.5–1 mg/kg/day elemental zinc orally for 3–4 months.
Vitamin D	Patients at risk of depletion or deficiency; severe kidney or liver dysfunction; prolonged immobilization or chronic illness; obesity; inflammatory bowel disease; bariatric surgery; pancreatic insufficiency; chronic intestinal failure; pregnancy; older age.	Serum 25-hydroxyvitamin D [25(OH)D]. Interpret considering inflammation and seasonal variation. Reassess at least once after 3–6 months; monitoring frequency should reflect deficiency severity and treatment dose.	EN: ≥1000 IU (25 μg)/day with 1500 kcal. PN: ≥200 IU (5 μg)/day. Recurrent deficiency: 4000–5000 IU (100–125 μg)/day for 2 months; target 25(OH)D 40–60 ng/mL. Higher doses may be required in selected patients.
Vitamin K1	Steatorrhea or fat malabsorption; prolonged broad-spectrum antibiotic therapy; chronic kidney disease; malnutrition; vitamin K antagonist therapy.	Dietary intake plus a combination of biomarkers; no single reference standard. Plasma phylloquinone, PIVKA-II, and coagulation tests may be considered. No fixed monitoring interval specified.	EN: ≥120 μg/day with 1500 kcal. PN: may provide 150 μg/day. Include vitamin K supplied by lipid emulsions in total intake. During vitamin K antagonist therapy, maintain a stable intake and monitor coagulation; withhold continuous EN for 1 h before and after anticoagulant administration.
Coenzyme Q10	No routine clinical indication for assessment; measurement is mainly limited to research settings.	Plasma CoQ10 for research purposes. No clinical monitoring interval specified.	No established DRI/RDA and no ESPEN recommendation for routine dosing or supplementation. No IV formulation currently available.
L-carnitine	Unexpected loss of lean body mass with concomitant hypertriglyceridemia and hyperlactatemia, particularly during prolonged PN or CRRT	Total and free carnitine, carnitine esters, and precursors; calculate the acyl-to-free carnitine ratio. Testing should confirm the clinical diagnosis but should not delay supplementation.	No routine addition to EN or PN. Proven deficiency: 2–5 mg/kg/day via the same route used for macronutrient administration until carnitine levels and the acyl-to-free ratio normalize. Antiretroviral drug toxicity: 50–100 mg/kg/day.
Selenium	PN expected for >2 weeks or initiation of home PN; prolonged EN; reduced prior intake; major burns or trauma; CRRT or other ongoing selenium losses.	Plasma selenium with CRP and albumin; ideally plasma GPX-3 for functional status. Measure at PN initiation; repeat according to results and at least every 3–6 months. Reassess after 7–10 days of high-dose IV correction.	EN: 50–150 μg/day with 1500 kcal. PN: 60–100 μg/day. Plasma selenium < 0.4 μmol/L (<32 μg/L): start 100 μg/day enterally or IV. Plasma selenium < 0.75 μmol/L with CRP < 20 mg/L: supplementation indicated. Severe deficiency: up to 400 μg IV/day for at least 7–10 days, followed by reassessment.
Copper	Previous bariatric or other surgery excluding the duodenum; unexplained neuropathy; major burns; CRRT > 2 weeks; home EN via jejunostomy; long-term PN.	Plasma copper with CRP; ceruloplasmin if results are uncertain. Every 6–12 months during long-term PN; more frequently with cholestasis.	EN: 1–3 mg/day with 1500 kcal. PN: 0.3–0.5 mg/day. Plasma copper < 12 μmol/L with CRP >20 mg/L: deficiency likely; consider supplementation. Plasma copper < 8 μmol/L: repletion indicated regardless of CRP. Severe deficiency: 4–8 mg/day IV by slow infusion; oral administration may be considered first in chronic conditions.
Vitamin B6 (pyridoxine)	Clinical signs of deficiency; alcohol use disorder; dialysis or CRRT; older age; postoperative state; infection or critical illness; pregnancy; treatment with isoniazid, penicillamine, corticosteroids, anticonvulsants, or anticancer drugs.	Plasma pyridoxal phosphate (PLP); red-cell PLP in severe illness or inflammation. PLP response generally plateaus after 6–10 days.	EN: ≥1.5 mg/day with 1500 kcal. PN: 4–6 mg/day. Isoniazid overdose: high-dose pyridoxine. Ethylene glycol poisoning: 50 mg IV every 6 h.
Vitamin B12 (cobalamin)	Anemia; isolated macrocytosis; established polyneuropathy; neurodegenerative disease or psychosis. Increased risk with vegan diets, older age, gastric or ileal surgery, bariatric surgery, malabsorptive disorders, metformin, or prolonged acid-suppressive therapy.	At least two biomarkers, preferably holotranscobalamin and methylmalonic acid; serum cobalamin may replace holotranscobalamin. Test anti-intrinsic factor antibodies in patients with autoimmune disease or glossitis, anemia, and neuropathy. Assess clinical and biochemical response at least annually in at-risk or treated patients.	EN: ≥2.5 μg/day with 1500 kcal. PN: ≥5 μg/day. Compromised absorption: lifelong 350 μg/day orally or 1000–2000 μg IM every 1–3 months. Acute symptomatic deficiency, pernicious anemia, total gastrectomy, or persistent malabsorption: 1000 μg IM every other day for 2 weeks or daily for 5 days; continue at least twice monthly until clinical resolution. Monitor serum potassium during repletion.

Abbreviations: BSA, body surface area; CRP, C-Reactive Protein; CRRT, continuous renal replacement therapy; DRI, dietary reference intake; EN, enteral nutrition; GPX-3, glutathione peroxidase 3; IM, intramuscular; IV, intravenous; PN, parenteral nutrition; PIVKA-II, protein induced by vitamin K absence or antagonist-II; PLP, pyridoxal phosphate; RDA, recommended dietary allowance.

## Data Availability

No new data were created or analyzed in this study. Data sharing is not applicable to this article.

## Supplementary Materials

The following supporting information can be downloaded at: https://www.mdpi.com/article/10.3390/jcm15156066/s1, Supplementary Material S1: PubMed Search String.

## Abbreviations

The following abbreviations are used in this manuscript:

ASMI	Appendicular Skeletal Muscle Mass Index
ASPEN	American Society of Parenteral and Enteral Nutrition
CIM	Critical Illness Myopathy
CoQ10	Coenzyme Q10
COVID	Coronavirus Disease
CRP	C-Reactive Protein
CRRT	Continuous Renal Replacement Therapy
ESICM	European Society of Intensive Care Medicine
ESPEN	European Society of Parenteral and Enteral Nutrition
ICU	Intensive Care Unit
ICU-AW	Intensive Care Unit-Acquired Weakness
ID	Iron Deficiency
MR	Mendelian Randomization
PLP	Pyridoxal Phosphate
RCT	Randomized Controlled Trial
WHO	World Health Organization
