# Supplementary material for: Micronutrients and ICU-Acquired Weakness: A Narrative Review
Source: J Clin Med. 2026 Aug 4;15(15):6066. doi: 10.3390/jcm15156066 (PMC13467528; doi:10.3390/jcm15156066)
Supplement: Supplementary file 1 [file jcm-15-06066-s001.zip › jcm-4444652-supplementary.pdf]

Micronutrients and ICU-Acquired Weakness: A narrative review.

Supplementary Material S1: PubMed Search string.

Search concept 1: Micronutrients

(("micronutrients"[Pharmacological Action]) OR ("micronutrients"[MeSH Terms]) OR ("micronutrients"[All Fields]) OR ("micronutriments"[All Fields]) OR ("trace elements"[Pharmacological Action]) OR ("trace elements"[MeSH Terms]) OR ("trace"[All Fields] AND "elements"[All Fields]) OR ("trace elements"[All Fields]) OR ("micronutrient"[All Fields]) OR ("Dietary Supplements"[Mesh]) OR ("Vitamins"[Mesh]) OR (“Vitamins” [All Fields]) OR (“Selenium” [All Fields]) OR ("Selenium"[Mesh]) OR (“Zinc” [All Fields]) OR ("Zinc"[Mesh]) OR ("Manganese"[Mesh]) OR (“Manganese” [All Fields]) OR ("Vitamin D"[Mesh]) OR (“Vitamin D” [All Fields]) OR ("Ascorbic Acid"[Mesh]) OR (“Ascorbic Acid” [All Fields]) OR (“Vitamin C” [All Fields]) OR ("Vitamin B Complex"[Mesh]) OR ("Vitamin B Complex" [Pharmacological Action]) OR ("Vitamin B Deficiency"[Mesh]) OR (“Vitamin B Deficiency” [All Fields]) OR ("Carnitine"[Mesh]) OR (“Carnitine” [All Fields]) OR ("Copper"[Mesh]) OR (“Copper” [All Fields]) OR (“Cuprum” [All Fields]) OR ("Iron"[Mesh]) OR (“Iron” [All Fields]) OR ("Vitamin K 2"[Mesh]) OR (“Vitamin K 2” [All Fields]) OR (“Vitamin K2” [All Fields]) OR (“Menaquinone” [All Fields]) OR ("Vitamin K"[Mesh]) OR (“Vitamin K” [All Fields]) OR (“Phylloquinone” [All Fields]) OR (“Vitamin K3” [All Fields]) OR (“Vitamin K 3” [All Fields]) OR ("Vitamin K 3"[Mesh]) OR ("Cadmium"[Mesh]) OR (“Cadmium” [All Fields]) OR ("Choline"[Mesh]) OR (“Choline” [All Fields]) OR ("Chromium"[Mesh]) OR (“Chromium” [All Fields]) OR ("Cobalt"[Mesh]) OR (“Cobalt” [All Fields]) OR ("Fluorides"[Mesh]) OR (“Fluoride” [All Fields]) OR ("Iodine"[Mesh]) OR (“Iodine” [All Fields]) OR ("Molybdenum"[Mesh]) OR (“Molybdenum” [All Fields]) OR ("Thiamine"[Mesh]) OR (“Thiamine” [All Fields]) OR (“Vitamin B1” [All Fields]) OR (“Vitamin B 1” [All Fields]) OR ("Riboflavin"[Mesh]) OR (“Riboflavin” [All Fields]) OR (“Vitamin B2” [All Fields]) OR (“Vitamin B 2” [All Fields]) OR ("Niacin"[Mesh]) OR (“Niacin” [All Fields]) OR (“Vitamin B3” [All Fields]) OR (“Vitamin B 3” [All Fields]) OR ("Pantothenic Acid"[Mesh]) OR (“Pantothenic Acid” [All Fields]) OR (“Vitamin B5” [All Fields]) OR (“Vitamin B 5” [All Fields]) OR ("Pyridoxine"[Mesh]) OR (“Pyridoxine” [All Fields]) OR (“Vitamin B6” [All Fields]) OR (“Vitamin B 6” [All Fields]) OR ("Biotin"[Mesh]) OR (“Biotin” [All Fields]) OR (“Vitamin B7” [All Fields]) OR (“Vitamin B 7” [All Fields]) OR ("Folic Acid"[Mesh]) OR (“Folic Acid” [All Fields]) OR (“Folate” [All Fields]) OR (“Vitamin B9” [All Fields]) OR (“Vitamin B 9” [All Fields]) OR ("Vitamin B 12"[Mesh]) OR (“Cobalamin” [All Fields]) OR (“Vitamin B 12” [All Fields]) OR (“Vitamin B12” [All Fields]) OR ("Vitamin A"[Mesh]) OR (“Vitamin A” [All Fields]) OR (“Retinol” [All Fields]) OR ("Vitamin E"[Mesh]) OR (“Vitamin E” [All Fields]) OR (“Tocopherol” [All Fields]) OR ("Ubiquinone"[Mesh]) OR (“Ubiquinone” [All Fields]) OR (“Coenzyme Q10” [All Fields]))

Search concept 2: Critical illness and intensive care

AND (("intensive care units"[MeSH Terms]) OR ("intensive"[All Fields] AND "care"[All Fields] AND "units"[All Fields]) OR ("intensive care units"[All Fields]) OR ("icu"[All Fields]) OR (“Critical Care” [All Fields]) OR ("Critical Illness"[Mesh]) OR ("Critical Care"[Mesh]) OR (“Critically Ill” [All Fields]))

Search concept 3: ICU-acquired weakness

AND (("Sarcopenia"[Mesh]) OR ("Muscle Weakness"[Mesh]) OR ("Immobilization"[Mesh]) OR ("Sarcopenia" [All Fields]) OR ("ICUAW" [All Fields]) OR ("ICU-AW" [All Fields]) OR ("Intensive Care Unit Acquired Weakness" [All Fields]) OR ("Muscle Weakness" [All Fields]) OR ("Muscle Wasting" [All Fields]) OR ("Muscle Dysfunction" [All Fields]) OR ("Muscle" [All Fields]) OR ("Skeletal Muscle" [All Fields]) OR ("Muscle, Skeletal"[Mesh]) OR ("Cachexia" [All Fields]) OR ("Muscular Atrophy"[Mesh]) OR ("Muscle Atrophy" [All Fields]) OR ("Muscular Atrophy" [All Fields]) OR ("postintensive care syndrome" [Supplementary Concept]) OR ("Post-intensive care syndrome" [All Fields]) OR ("Post intensive care syndrome" [All Fields]) OR ("Postintensive care syndrome" [All Fields]))

Complete search string:

Search: **(("micronutrients"[Pharmacological Action]) OR ("micronutrients"[MeSH Terms]) OR ("micronutrients"[All Fields]) OR ("micronutriments"[All Fields]) OR ("trace elements"[Pharmacological Action]) OR ("trace elements"[MeSH Terms]) OR ("trace"[All Fields] AND "elements"[All Fields]) OR ("trace elements"[All Fields]) OR ("micronutrient"[All Fields]) OR ("Dietary Supplements"[Mesh]) OR ("Vitamins"[Mesh]) OR ("Vitamins" [All Fields]) OR ("Selenium" [All Fields]) OR ("Selenium"[Mesh]) OR ("Zinc" [All Fields]) OR ("Zinc"[Mesh]) OR ("Manganese"[Mesh]) OR ("Manganese" [All Fields]) OR ("Vitamin D"[Mesh]) OR ("Vitamin D" [All Fields]) OR ("Ascorbic Acid"[Mesh]) OR ("Ascorbic Acid" [All Fields]) OR ("Vitamin C" [All Fields]) OR ("Vitamin B Complex"[Mesh]) OR ("Vitamin B Complex" [Pharmacological Action]) OR ("Vitamin B Deficiency"[Mesh]) OR ("Vitamin B Deficiency" [All Fields]) OR ("Carnitine"[Mesh]) OR ("Carnitine" [All Fields]) OR ("Copper"[Mesh]) OR ("Copper" [All Fields]) OR ("Cuprum" [All Fields]) OR ("Iron"[Mesh]) OR ("Iron" [All Fields]) OR ("Vitamin K 2"[Mesh]) OR ("Vitamin K 2" [All Fields]) OR ("Vitamin K2" [All Fields]) OR ("Menaquinone" [All Fields]) OR ("Vitamin K"[Mesh]) OR ("Vitamin K" [All Fields]) OR ("Phylloquinone" [All Fields]) OR ("Vitamin K3" [All Fields]) OR ("Vitamin K 3" [All Fields]) OR ("Vitamin K 3"[Mesh]) OR ("Cadmium"[Mesh]) OR ("Cadmium" [All Fields]) OR ("Choline"[Mesh]) OR ("Choline" [All Fields]) OR ("Chromium"[Mesh]) OR ("Chromium" [All Fields]) OR ("Cobalt"[Mesh]) OR ("Cobalt" [All Fields]) OR ("Fluorides"[Mesh]) OR ("Fluoride" [All Fields]) OR ("Iodine"[Mesh]) OR ("Iodine" [All Fields]) OR ("Molybdenum"[Mesh]) OR ("Molybdenum" [All Fields]) OR ("Thiamine"[Mesh]) OR ("Thiamine" [All Fields]) OR ("Vitamin B1" [All Fields]) OR ("Vitamin B 1" [All Fields]) OR ("Riboflavin"[Mesh]) OR ("Riboflavin" [All Fields]) OR ("Vitamin B2" [All Fields]) OR ("Vitamin B 2" [All Fields]) OR ("Niacin"[Mesh]) OR ("Niacin" [All Fields]) OR ("Vitamin B3" [All Fields]) OR ("Vitamin B 3" [All Fields]) OR ("Pantothenic Acid"[Mesh]) OR ("Pantothenic Acid" [All Fields]) OR ("Vitamin B5" [All Fields]) OR ("Vitamin B 5" [All Fields]) OR ("Pyridoxine"[Mesh]) OR ("Pyridoxine" [All Fields]) OR ("Vitamin B6" [All Fields]) OR ("Vitamin B 6" [All Fields]) OR ("Biotin"[Mesh]) OR ("Biotin" [All Fields]) OR ("Vitamin B7" [All Fields]) OR ("Vitamin B 7" [All Fields]) OR ("Folic Acid"[Mesh]) OR ("Folic Acid" [All Fields]) OR ("Folate" [All Fields]) OR ("Vitamin B9" [All Fields]) OR ("Vitamin B 9" [All Fields]) OR ("Vitamin B 12"[Mesh])**

OR ("Cobalamin" [All Fields]) OR ("Vitamin B 12" [All Fields]) OR ("Vitamin B12" [All Fields]) OR ("Vitamin A"[Mesh]) OR ("Vitamin A" [All Fields]) OR ("Retinol" [All Fields]) OR ("Vitamin E"[Mesh]) OR ("Vitamin E" [All Fields]) OR ("Tocopherol" [All Fields]) OR ("Ubiquinone"[Mesh]) OR ("Ubiquinone" [All Fields]) OR ("Coenzyme Q10" [All Fields])) AND (("intensive care units"[MeSH Terms]) OR ("intensive"[All Fields] AND "care"[All Fields] AND "units"[All Fields]) OR ("intensive care units"[All Fields]) OR ("icu"[All Fields]) OR ("Critical Care" [All Fields]) OR ("Critical Illness"[Mesh]) OR ("Critical Care"[Mesh]) OR ("Critically Ill" [All Fields])) AND ("Humans"[Mesh]) AND (("Sarcopenia"[Mesh]) OR ("Muscle Weakness"[Mesh]) OR ("Immobilization"[Mesh]) OR ("Sarcopenia" [All Fields]) OR ("ICUAW" [All Fields]) OR ("ICU-AW" [All Fields]) OR ("Intensive Care Unit Acquired Weakness" [All Fields]) OR ("Muscle Weakness" [All Fields]) OR ("Muscle Wasting" [All Fields]) OR ("Muscle Dysfunction" [All Fields]) OR ("Muscle" [All Fields]) OR ("Skeletal Muscle" [All Fields]) OR ("Muscle, Skeletal"[Mesh]) OR ("Cachexia" [All Fields]) OR ("Muscular Atrophy"[Mesh]) OR ("Muscle Atrophy" [All Fields]) OR ("Muscular Atrophy" [All Fields]) OR ("postintensive care syndrome" [Supplementary Concept]) OR ("Post-intensive care syndrome" [All Fields]) OR ("Post intensive care syndrome" [All Fields]) OR ("Postintensive care syndrome" [All Fields]))
